# The contribution of political skill to the implementation of health services change: a systematic review and narrative synthesis

**DOI:** 10.1186/s12913-021-06272-z

**Published:** 2021-03-20

**Authors:** Jenelle M. Clarke, Justin Waring, Simon Bishop, Jean Hartley, Mark Exworthy, Naomi J. Fulop, Angus Ramsay, Bridget Roe

**Affiliations:** 1 School of Social Policy, HSMC, University of Birmingham, Park House, 40 Edgbaston Park Road, Birmingham, B15 2RT UK; 2 Business School North, University of Nottingham, Jubilee Campus, Triumph Road, Nottingham, NG8 1BB UK; 3 Open University Business School, Open University, Walton Hall, Kents Hill, Milton Keynes, MK7 6BH UK; 4 Department of Applied Health Research, University College London, 1-19 Torrington Place, London, WC1E 7HB UK

**Keywords:** Political skill, Health services research, Health system change, Health organisation

## Abstract

**Background:**

The implementation of strategic health system change is often complicated by informal ‘politics’ in healthcare organisations. Leadership development programmes increasingly call for the development and use of ‘political skill’ as a means for understanding and managing the politics of healthcare organisations. The primary purpose of this review is to determine how political skill contributes to the implementation of health services change, within and across organisations. The secondary purpose is to demonstrate the conceptual variations within the literature.

**Methods:**

The article is based upon a narrative synthesis that included quantitative, qualitative and mixed methods research papers, review articles and professional commentaries that deployed the concept of political skill (or associated terms) to describe and analyse the implementation of change in healthcare services.

**Results:**

Sixty-two papers were included for review drawn from over four decades of empirically and conceptually diverse research. The literature is comprised of four distinct literatures with a lack of conceptual coherence. Within and across these domains, political skill is described as influencing health services change through five dimensions of leadership: personal performance; contextual awareness; inter-personal influence; stakeholder engagement, networks and alliances; and influence on policy processes.

**Conclusion:**

There is a growing body of evidence showing how political skill can contribute to the implementation of health services change, but the evidence on explanatory processes is weak. Moreover, the conceptualisation of political skill is variable making comparative analysis difficult, with research often favouring individual-level psychological and behavioural properties over more social or group processes.

**Supplementary Information:**

The online version contains supplementary material available at 10.1186/s12913-021-06272-z.

## Background

The implementation of organisational change within health and care services is notoriously difficult. Of the many contextual factors shown to influence change processes there is growing, but still relatively limited, recognition that organisational politics can have a significant bearing on the implementation of change. Although more formal (big ‘P’) political institutions and policy-making processes are commonly studied as triggering or shaping health services change, the more informal (small ‘p’) politics of care services can also influence change processes. Bate et al. [1] illustrate the ‘political challenge’ of implementing quality improvement in terms of securing buy-in, dealing with conflict, building relationships and agreeing a common agenda. In similar ways, the burgeoning field of implementation science highlights how stakeholders’ divergent interests can shape how new models of service organisation are implemented and sustained [2, 3]).

Growing recognition of the informal politics of healthcare services has led to corresponding calls for health and care leaders to acquire and utilise a distinct set of skills and capabilities specifically tailored to understanding and dealing with the ‘political arenas’ of health service change. Pre-dating the current focus on health and care leaders, research since the mid-1970s called for members of the nursing profession to develop forms of political astuteness when seeking to influence policy-making [4]. More recently, Montalvo’s [5] integrative review of the nursing literature shows that ‘political skill’ can enhance nurses’ career development and impact on group performance through increased personal resilience, inter-personal influence, and influence within organisational networks. Similarly, Gilson [6] calls for greater attention to organisational politics and political skill in healthcare leadership development so that leaders are better equipped to understand the multiple agendas and interests that impact on everyday service organisation. Such ideas have now become integral to formal leadership development programmes. The ‘Leadership Qualities Framework’ used by the United Kingdom (UK) National Health Service Leadership Academy [7] emphasised the importance of ‘political astuteness’ in terms of a) the capacity to understand the climate and culture of the organisation; b) knowing who the key influencers are and how to involve them; c) being attuned to national and local strategies; and d) understanding the inter-connected roles of leadership. Although reviews in the nursing field provide some evidence for these skills in terms of nursing objectives and agendas, there is wide variation in the definition and conceptualisation of political skill, astuteness, savvy and intelligence, as well as variations in how such concepts can help inform or explain the implement of health service change.

Developed alongside the literature on healthcare politics, but as we show, with only partial cross-fertilisation, the management and leadership studies literatures offer a well-developed source of theoretical and empirical understanding of organisational politics and political skill. Pfeffer ([8],p7) describes such politics as “… *those activities taken within organizations to acquire, develop, and use power and other resources to obtain one’s preferred outcomes in a situation in which there is uncertainty or dissensus about choice*”. He also describes the importance of leaders utilising ‘political skills’ to manage with and through politics, including ‘political strategies’ to control the agenda, build coalitions and co-opt resistant groups; ‘political language’ to frame ideas, shaping meaning and persuade others; and ‘controlling resources’ to dis/able activities or incentivise change. In recent years, the concept of political skill has been developed by Ferris and colleagues who define it as the “… *ability to effectively understand others at work, and use this understanding to influence others to act in ways that enhances one’s personal and/or organizational objectives*” [9,p127]. This is elaborated along four dimensions, including social astuteness (the ability to observe situations and adjust behaviours accordingly), inter-personal influence (the ability to change the behaviours of others), networking ability (having access to information and resources through connections), and apparent sincerity (to be perceived as having integrity). A recent review of this broader literature shows that a range of further definitions and concepts are used to describe the different facets of political astuteness, intelligence, and savvy [9, 10].

Although there is little doubt health and care services are inherently political and people act politically in the organisation of care, it is important to clarify the conceptual premise of ‘political’ and ‘politics’ before considering the specific concept of ‘political skill’, especially as this clarification can provide a basis for conceptual critique and extension. First, we draw on the work of Carl Schmitt [11], whose suggests that social groups are distinguished by distinct beliefs, values and ideologies that represent their underlying ‘political’ differences, and from which groups come to be regarded as ‘friend or foe’. These existential ‘political’ differences provide the basis of a political community motivated to engage in political activities, which become the substance of ‘politics’ whether in the form of judicial-political systems, or other social or organisational processes. The substance of politics can therefore involve multiple ‘arenas’ and ‘processes’ through which such political differences are articulated; the form of which clearly varies between time and culture.

From this broad conceptualisation two further points of clarification are relevant to the study of organisational politics. The first is around the interests and agenda that define and distinguish social groups. As shown below, much of the writing on organisational politics and political skill stems from organisational psychological and tends to portray interests and agenda in relatively narrow or self-serving terms, i.e. people engage in organisational politics for their own advancement [12]. In contrast, more critical sociology and public policy research tends to see interests as more cultural, social and structural in character, being acquired through socialisation and reflecting social institutions and ideological imperatives [13] for example shared professional agendas [14]. Rather than reducing analysis to either position (agency or structure), this research is attentive the interplay between the two; for example, where engaging in organisational politics can be driven to advance the personal advancement of the professional leader, the collective agenda of a profession, and the ideological imperatives of professionalism.

The second point of clarification deals with the ontology of organisational politics and political skill. It is noteworthy that the term ‘micro-politics’ is also used to describe this aspect of organising, which more clearly indicates a concern with ‘micro-level’ actions and interactions. From this perspective, ‘micro-politics’ is not confined to a particular organisational or social hierarchy, i.e. shopfloor or board room, but rather to the level of actions and interactions, whether on ward or board; whilst recognising these are situated within particular social and cultural contexts. However, the term ‘micro-politics’ (more than organisational politics) inevitably implies some notion of ‘macro’, and returning to the above discussion of interests, this notion of ‘macro’ does not deal with the hierarchies of organising or policy-making (i.e. national politics), rather the term macro is used in a more sociological sense of being institutional, structural and societal. This therefore connects the idea of macro structural or political interests to the micro actions and interactions of organisational politics; in ways that is less obvious in much of the literature on ‘organisational politics’. As such, the concept micro-politics focuses on the micro-level actions through which different interests and agendas are played out, recognising that these interests can be both self-serving and also structural interests.

The primary purpose of this article is to review the health service research literature to determine how political skill contributes to the implementation of health services change. The secondary purpose is to demonstrate the conceptual and methodological variations within the health services research literature and to understand how these can lead to different interpretations of change.

## Method

### Narrative review

Given that the topic under review was known to comprise diverse theoretical, methodological and empirical orientations, a systematic review with a narrative synthesis methodology was adopted [15–17]. Rather than seeking to synthesize and analyse statistical results, as in a traditional systematic review, a systematic review with a narrative synthesis aims to develop a thematic summary of multiple diverse research sources involving interpretation and critique. Although sometimes criticized for selecting judicious sources, narrative synthesis reviews are not necessarily un-systematic but, as described below, followed a step-wise and transparent approach [18].

### Search strategy

A preliminary task involved clarifying the search terms for ‘political skill’ and the boundaries of the ‘health services research’ literature. As described above, defining what is meant by ‘political’ within healthcare is challenging, and our focus is concerned with the micro-level politics. Within the management studies field, a number of conceptual definitions define the field, such as political ‘skill’, ‘astuteness’, ‘savvy’ or ‘intelligence’, although the concept developed by Ferris and colleagues [19, 20] over the last decade has become the more prominent. For the purpose of this review, an explicitly inclusive approach was taken to consider the variety of ways political skill has been deployed within the health services research literature to understand the implementation of service change, inclusive of terms found within the wider management literature. Nonetheless, by focusing on ‘political’ in our search strategy, we recognise that our review is dependent upon authors choosing to label their organisational phenomena as political. The field of ‘health services research’ was defined following the *Association for Health Services Research* and the *Academy of Health Services Research and Health Policy*, which describe it as a multidisciplinary field that studies how ‘social factors, financing systems, organizational structures and processes, health technologies and personal behaviours affect access to health care, the quality and cost of health care and ultimately … health and well-being’ ([21],p16).

Taking into account the above clarifications, the search and selection criteria were refined through a deliberative process amongst all authors and a panel of research experts drawn from the fields of health services research, public policy, organisation studies, health psychology and medical sociology (*n* = 8). Through this process we established the broad parameters of our search in terms of concepts that describe and explain how organisational actors develop and use particular skills, behaviours or strategies to understand, influence or manage the informal political context of their organisation or workplace. Working with the expert panel, the following search terms, Boolean operators were identified: political skill OR political astuteness OR political savvy OR political acumen OR political nous OR socio-political intelligence OR political leadership AND health OR healthcare OR health service OR health policy OR health policies (see Additional file 1: Appendix 1 for a search example). Using these search terms, a systematic literature search was undertaken using seven well-established databases to offer coverage across the health services research literatures: MEDLINE*,* Web of Science*,* PsychInfo*,* ProQuest Social Science, PubMed, CINAHL Plus, SCOPUS. No time restrictions were placed. The searches were run between October and November 2018. We also carried out hand searches based on consultation with the expert panel, and through reviewing the bibliographies and references of included literature for further sources, and literature recommended by domain-relevant experts. In total, the database and manual searches identified 1718 records.

**Fig. 1:**
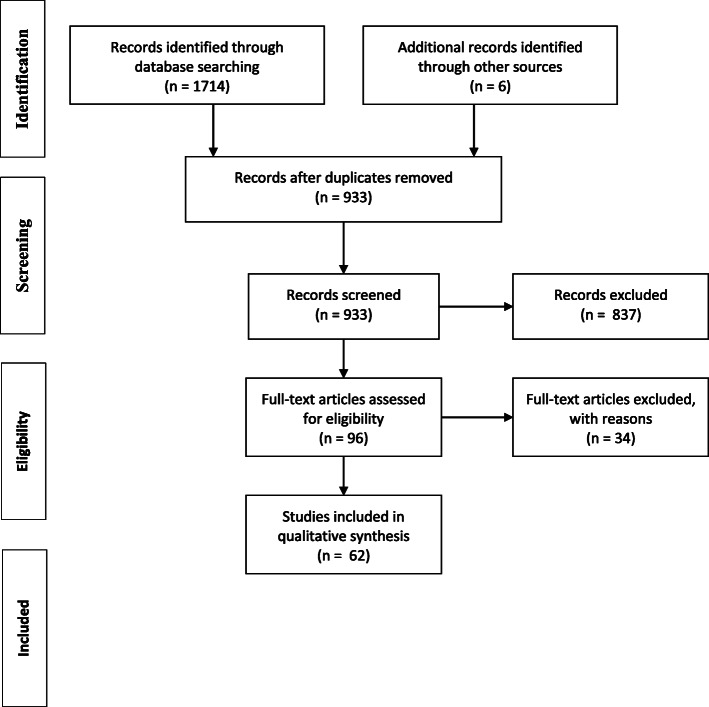
PRISMA Flow Diagram [79]

The primary basis of inclusion was whether an article explicitly deployed and/or reported on political skill (or similar term) as part of research addressing the implementation of change within the organisation of healthcare services. The review was inclusive of empirical and theoretical papers, evidence-based commentaries, and prominent grey literature. All included papers were written in English. No time restrictions were applied. Three independent reviewers (anonymized for review) screened the results (titles and abstract review), excluding 837 papers. Two authors then independently reviewed 96 articles (full text), excluding a further 35, with 62 identified [5, 6, 9, 10, 22–78] for inclusion (Fig. 1) [80].

Where disagreements between reviewers occurred, authors deliberated viewpoints with the third reviewer contributing to the selection process.

### Extraction and analysis

A standardized spreadsheet was used to extract relevant data thematically [80, 81]. Characteristics included: the full citation, method of study, disciplinary perspective, theoretical background, phenomenon of interest in regard to the change agenda or political issues, context for study, methodological position, analytical approach, key findings, interpretation and explanation related to the study of change, and theoretical contribution. Extracted information was discussed on a regular basis between all authors. Preliminary findings were presented to the full team and panel of experts for further refinement. Narrative analysis of the papers involved interpretation of prominent themes within and across the selected literature. First, the structure of the literature was summarised in terms of main disciplinary fields and areas of study, which related to distinct policy context and professional domain over a four-decade period. Second, analysis focused on how political skill or related concepts was shown to contribute to the implementation of health services change, identifying the main explanatory accounts along five thematic lines: personal performance; contextual awareness; inter-personal influence; stakeholder engagement, networks and alliances; and influence on policy processes. Supplementary analysis considered the different theoretical and methodological approaches informing health services research. Following Millar et al. [15], Popay et al. [80] and Dixon-Woods et al. [81], we prioritized papers along two lines of enquiry related to our review questions: quality and relevance (Table 1).

**Table 1:** Influential Studies and Papers Exploring Political Skill within Healthcare Settings

Author(s)	Type	Disciplinary Context	Aims	Methods	Summary of Findings
Benton et al. [30]	Review	Nurses	Integrative review of papers exploring nurses’ involvement in policy and political processes	Integrative review methods to identify relevant studies in English between 1965 and 2015. Use of comparative thematic analysis.	45 studies identified, mostly in North America, mostly descriptive surveys with small sample sizes. Papers focused on developing nurses’ awareness of legislative and political processes. Literature ignores political awareness at team and organizational level and informal-politics largely ignored.
Berger [61]	Conceptual	Social workers	To identify social workers’ power, including political acumen, within hospitals	Not applicable	Advocates for social workers developing political skills through awareness of power sources at individual and organizational level, particularly through maximizing their position within the hospital power network (e.g. through coordinating discharge and case management).
Byrd et al. [23]	Empirical	Nurses	Assessing changes in nursing baccalaureate students’ participation in public policy learning activities	Use of the Political Astuteness Inventory with 300 nursing students before and after completion in the learning activities	Political astuteness scores significantly increased after participation in the learning activities. Active learning and increased political astuteness can increase knowledge of health policy.
Comber et al. [56]	Empirical	Physicians	To understand physicians’ leadership of clinical and non-clinical roles through assessing their political skill (as defined by Ferris et al. (2005)).	Sample of 209 Canadian physicians using the Political Skills Inventory	Physicians in clinical roles had significantly lower PSI scores compared with those in non-clinical roles. Authors advocate for further training of political skills for physicians in clinical roles.
Crow and Hartman [34]	Conceptual	Managers	Addressing the political skill gap for healthcare managers, with suggestions for how to acquire and improve political skill	Not applicable	The authors highlight that managers can feel intimated by those with higher education degrees and advocate the use of different powers, including the ability to read situations accurately and using coercion and influence.
Gilson [6]	Theoretical, drawing on empirical	Systems	Reflections on everyday politics of healthcare systems, relevance for healthcare policy, and leadership skills required to navigate politics	Not applicable	Everyday politics within health systems involve multiple actors and agendas that frontline leaders must address that impacts upon how groups take action throughout the system. New forms of leadership training are required to develop political leadership skills.
Greer and Lillvis [73]	Review	Public health	Joins Health in All Policies with political science literature to explore difficulties with coordinating initiatives across government sectors.	Review is theoretically driven with few details on methods	Two challenges identified: coordinating initiatives across different sectors and sustaining initiatives. Authors advocate not relying solely on political leadership but use outside ‘allies’ and networks to influence policy.
Hunter et al. [82]	Empirical	Systems	Examination of the North East transformation system in the NHS, including its formation, progress and changes as result of government policy.	Longitudinal 3.5 year study using mixed methods with 14 NHS Trusts in the North East, includes 68 interviews, observations and focus groups.	Politics in system change is often ignored in research. Implementing whole system change is subject to changes in politics, especially the NHS which is itself influenced by wider government policies.
McAuliffe et al. [70]	Empirical	Systems	Identify the factors associated with reforms and change of the Irish cancer service	In-depth retrospective analytical case study exploring the implementation of the 8 Irish breast cancer services and interviews with stakeholders	Key success to implement and sustain change include: timing, involvement of stakeholders/public support, framing the necessity for the change, clarifying scope of change, support of political leadership.
Montalvo and Byrne [25]	Empirical	Nurses	To analyze the link between mentoring and political skill development with nurses who had earned, or were studying for, a PhD in nursing or doctorate of nursing practice.	Web based survey, including the Political Skill Inventory (developed by Ferris and colleagues) with 222 nurses who had, or were working towards, a PhD in nursing or doctorate of nursing practice.	Mentoring was significant for developing political skill and mentorship arrangements (formal or informal) benefit nurses for acquiring political skill.
Montalvo [5]	Review	Nurses	Integrated analysis of political skill literature and relevance to nursing	Literature reviews and empirical papers from 2000 to 2014 that defined political skill as the ability to influence others at work for personal development or achieving organizational goals	Political skill helps individuals navigate organizational politics, influences their ability to network, manage stress, enhance their performance and increase interpersonal skills
Primomo [49]	Empirical	Nurses	Assessing political astuteness of nursing students before and after a course on health policy	Use of the Political Astuteness Inventory with 57 Masters of Nursing students before and after completion of the health policy course	Political astuteness scores significantly increased after completion of the course. Primomo advocates health policy education in order for nurses to influence healthcare change
Smaltz et al. [66]	Empirical	Hospital Chief Information Officers (CIOs)	Explores how CIOs can be effective in the healthcare sector	Surveys with 185 CIOs (Phase 1) and surveys with 136 individuals from top management team members (Phase 2)	Along with interpersonal skills and IT skills, political savvy had a significant positive relationship to the role effectiveness of a CIO. Developing trusting relationships with members of the top management team was significant.
Taylor [58]	Conceptual	Managers	Focuses on the political skills required for managers and administrators working within Canadian health settings	Not applicable	The political skill for different levels of managers are outlined, including the need to be self-aware, the ability to influence others, knowledge of interorganizational policies, examining the internal/external environments to identify key issues
Turner et al. [71]	Empirical	Systems	Identify factors that influence selection of acute stroke centralization models in London and Greater Manchester	Analysis of 316 documents and 45 interviews with those leading the system change	The combination of system and distributed leadership are most effective in implementing system change. Involving multiple stakeholders is crucial for those leading system change, along with a coordinating body with political authority to combine multiple interests.
Wilber and Coberly [59]	Empirical	Gerontologists	Determine the desired educational policy requirements for gerontologists	Surveys with 114 prospective gerontologist employers or those training gerontologists	Communication and political skill are essential for gerontologists working in policy arenas. Internships were considered desirable to acquire these skills.

## Results

### Structure of the literature

The review found that the health services research literature was structured along four lines when deploying the concept of political skill in the study of health services change, each reflecting different policy and professional contexts that have been the focus of research over the last four decades (Table 2).

**Table 2:** Thematic Summary of Reviewed Literature

Domain	Period	No. of Papers	Definition of Political Skill	Main Methods	Empirical topics and themes	Interpretative Analysis
Nursing	1970–2017	35	Largely pre-dates work of Ferris et al. [19] Nursing has legitimate role in health care decision making. But nurses lack formal power in relation to medicine and so require other approaches to influence (political skill as alternative to formal power). Definition rarely defined and is generalized.	Conceptual, surveys, some interviews, some ethnographies	Focus on nurses developing and using political skill or astuteness to secure greater influence in an organization, or more often to engage and have influence in legislative process	The concept of political skill linked to the macro- politics of nursing profession, especially the professionalization and politicizing agendas of US nursing. Some focus on workplace informal politics.
Healthcare Managers	1979–2018	9	Largely pre-dates work by Ferris et al. [19] Concept of political skill not always defined or explained theoretically. Managers should be leading organizational change and require political skill to rise above inter-professional politics (political skill as change tactic).	Conceptual, surveys, some interviews	Focus on how hospital managers (can) use political skill or other forms of inter-personal influence to implement organization change, with some attention given to external policy relations	The concept of political skill linked to the informal politics of organizational change and broader politics of management reform, especially the difficulties of managing change in context of professional resistance.
Other Health Professionals (including public health)	1991–2017	13	Conceptualizations similar to nursing Concept of ‘political skill’ not always defined or explained theoretically, exception of Hartley’s work on ‘political astuteness’	Conceptual, surveys, interviews	Focus on how non-nursing professionals use political skill to influence organization of work, especially in policy making processes, and through the professional leadership acting ‘politically’.	The concept of political skill seems to have been transferred from nursing with a focus on inter-personal influence or macro-influence on policy processes, dealing with professional dominance and more often influencing policy decisions.
System Leadership	1998–2016	5	Multiple stakeholders hold competing interests which need to be managed (political skill as form of negotiation) Concept of ‘political skill’ not defined or explained theoretically but descriptive aspects of the concept are linked to conceptions of system leadership	Surveys, interviews, ethnographies	Focus on how system leaders understand and manage competing interests in the implementation of large-scale system change.	The concept is aligned close with ‘system leadership’ and reflects broader shifts in analysis from managers to leaders and a single organization to a system.

Emerging in the 1970s, the first literature deals with nurse leaders’ use of political skill when seeking to influence formal policy processes. This literature reflects broader goals to enhance the professional status of nursing. From the 1980s, the second deals with the use of political skill by health service administrators, managers and, later, leaders in the implementation of organisational change. This literature coincides with the broader managerialisation of healthcare services throughout the 1980s; and shows growing influence of theories and models derived from the management studies literature. Earlier articles utilise a generic and un-theorised concept of political skill, but from the 1990s papers draw more explicitly on the work of Ferris and colleagues. The third (more dispersed) literature deals with the use of political skill by other (non-nursing) sections of healthcare workforce in the implementation of change. In many ways this mirrors the earlier research in the nursing field, and over time has become informed by various conceptualisations of political astuteness, intelligence and savvy, but with the work of Ferris and colleagues again becoming the more prominent. The fourth literature deals with the use of political skill in the context of more recent debates on ‘system leadership’ and the recognition that contemporary service change involves working with multiple stakeholders located within and across different organisational and occupational boundaries. Although political skill is rarely conceptualised in a formal way, research describes elements of system leadership as involving forms of political skill. The thematic review described below details the different dimensions and qualities of political skill (and associated terms) as deployed within the health services research literature to understand the implementation of health services change. This thematic review does not emphasise any particular prior theorisation of political skill, but does acknowledge where included research papers do draw on a particular conceptualisation.

### Thematic analysis

#### Personal performance

A relatively small number of research papers describe political skill as a form of personal competency, self-belief and self-efficacy that is strongly associated with enhanced personal performance and career development in the context of prevailing patterns of organisational politics (e.g. Montalvo [5], Taylor [58], Young [67], Lussier [64], Carroll [24]. The majority of these papers focus on nurse administrators and leaders and being initially based upon relatively generalised notions of political skill, but over time becoming informed by the conceptual work of Ferris et al. [19]. For example, Montalvo’s [5] integrative review of the nursing literature describes political skill as a form of personal mastery and security for navigating inter-personal relationships and influencing group dynamics in the workplace. This is associated with personal resilience, accumulated influence and career progression. The theme of resilience, coping and survival in the face of competing interests is also discussed in the parallel literature on hospital managers. Taylor [58] describes the importance of managers’ self-awareness or knowing one’s capabilities in the context of prevailing lines of power. Similarly, Young [67] describes the importance of laboratory managers developing personal routines and political style to deal with the political behaviours of others. Political skill is further invoked to explain different approaches to workforce management; for example, Lussier [64] focuses on the use of political skill to enhance job performance and advance personal agendas. Whitman et al.’s [55] surveys of nurse supervisors found that political skill can act as a self-regulatory mechanism to discourage supervisors from adopting assertive and abusive behaviours with subordinates. This theme therefore deals with the personal and psychological aspects of political skill, showing how these relate to other forms of inter-personal and organisational influence when implementing change.

#### Contextual awareness

A substantial theme within the literature describes political skill in terms of health service leaders’ ability to understand the prevailing political dynamics within their local service environment (e.g. Berger [61], Crow and Hartman [34]; Gilson [6]; Montalvo any Byrne [25]; Smaltz et al. [66], Taylor [58]). Three linked aspects of such contextual understanding are described in the literature. The first is to understand the prevailing ‘lines of power’ manifest amongst different stakeholders, especially which of these are more influential, dominant or likely to resist change [34, 58, 66]. The second aspect, although less developed in the literature, deals with leaders ability to understand the underlying interests or motivations of these groups [6]. As an example, Taylor [58] considers how health managers must use political skills to engage with wider community groups, especially when building networks around change agenda. The third aspect is to determine how best to respond to or manage these lines of power when seeking to implement change, i.e. to draw support from some groups and manage the resistance of others [61]. For instance, Berger [61] examines the need for social workers to gain awareness of power and politics within a hospital organisation in order to exert influence over patient management, particularly during discharge planning. Berger highlights how social workers use political skill to manage the competing interests amongst hospital doctors and nurses, and also carers and family members. She offers three recommendations for social workers: identifying power sources, successfully interpreting an organisation’s political environment, and using power effectively.

A number of studies describe this contextual awareness in terms of inter-professional working, i.e. understanding the power differences between nurse leaders, hospital administrators, senior executives and medical doctors (e.g. Montavlo [5, 44]; Montavlo and Byrne [25]; Berger [61]; Taylor [58]. That said, some studies similarly describe the need for medical leaders to utilise similar skills when implementing change, vis-a-vis other healthcare professionals and managers (Comber et al. [56]; Wilber and Coberly [59]). Reviewing this literature, research before the 1990s offers a relatively atheoretical, descriptive view of nurse leaders’ and hospital managers’ ability to ‘read situations’ and assess ‘lines of power’; whilst more recent research is more explicitly informed by Ferris et al.’s [19] idea of ‘social astuteness’ or similar concepts of ‘situational awareness’ drawn from the public management and organisational psychology literatures [9].

#### Inter-personal influence

Nearly all identified studies describe political skill as involving forms of inter-personal influence, but this was especially the case for studies or review articles that deployed the Ferris et al. [19] conceptualisation of political skill [5, 58, 66]. These papers tend to focus on ability of *Person A* to use particular inter-personal tactics to influence the behavioural responses of *Person B* in a given change context. For example, Smaltz et al. [67,p11] discuss how chief information officers need to develop political savvy, defined as the ability to ‘negotiate, influence and persuade’, in order to convince colleagues of information technology (IT) opportunities, to identify risks to projects delivery and to have more control over resources for IT projects. The studies describe inter-personal influence in a number of slightly different ways, such as persuasion, negotiation and coercion [5, 34, 61, 65]. However, the research studies rarely elaborate or theorise the specific types, features or boundaries of inter-personal influence.

The more recent literature of ‘system leadership’ highlights the importance of transformational and distributed leadership when seeking to engage and influence others. Turner et al.’s [71] study of major system change describes the importance of balancing an assertive or directive approach with an inclusive and delegated approach as two complementary approaches to inter-personal influence, i.e. where leaders set very clear parameters and expectations for change whilst also creating opportunities for engagement. Although this literature focus on different occupational relations (e.g. managers influencing doctors) and contexts (e.g. the implementation of information technology innovations), the underlying conceptualisation of political skill in relation to the implementation of change tends to emphasise individualised skills and abilities that are associated with individual psychology or capability and manifest in inter-personal influence, rather than more shared occupational or professional competence.

#### Stakeholder engagement, networks and alliances

Building on the themes of contextual awareness and inter-personal influence, the literature describes political skill in terms of a broader form of stakeholder engagement and network building. This is associated with the ability to understand and mediate the divergent interests of stakeholders in order to engage and align them positively with a given change agenda. McAuliffe et al.’s [70] study of health system change highlights the importance of stakeholder engagement as an element of more general change management activities, in which healthcare leaders should clarify the purpose, scope and timing of change in ways that aligns with the prevailing expectations of stakeholders. It also relates to a more prominent feature of the literature dealing with the importance of healthcare leaders’ communication strategies [59]. Again, the literature is very general in its description of these engagement and communication strategies, offering little in the way of detail on presentational style or framing techniques that might be used in the interactions between different professional groups. Mateo et al. [65] and Rafferty and Traynor [50] describe the importance of nurse leaders’ information processes (and stewardship) skills, especially for communicating national policy changes into local service contexts. Wilber and Coberly [59] similarly describe the importance of doctors’ different communication skills when working with internal and external stakeholders. In their study of major system change, Turner et al. [71] highlight the importance of using information systems and feedback loops to maintain stakeholder engagement during change processes. The ubiquity of communication and engagement strategies to healthcare management, in general, and change management, in particular, might account for the relative lack of empirical detail provided in the identified literature; although the wider health services research literature shows growing sophistication in its analysis of framing strategies but in ways that is not explicitly associated with political skill [2]. More recent research further demonstrates the importance of building networks and alliances at the inter-organisational level in the context of system change [58, 71, 73]. Although this still involves developing connections between clinical teams or departments the level of activity moves to the inter-organisational level, with commensurate recognition that hospitals and other healthcare organisations will often hold divergent priorities and drivers for strategic change that need to be reconciled and aligned with seeking reconfiguration of inter-agency care systems. This includes both reconciling differences between healthcare organisations such as in the centralisation of stroke services [71] and increasingly health and social care organisations [61].

Common to much of this research is the idea of an individual leader, often as part of group, extending their influence beyond the inter-personal level across clinical teams, professional communities or organisational departments. Although rarely made explicit, the assumption seems to be that change required forms of collective action that goes beyond the inter-personal influence described above, and therefore offering an important analytical shift in the conceptualisation of political skill (albeit one that is rarely made explicit in the literature).

#### Influence on policy processes

Lastly, extending beyond the arena of organisational politics, a significant and early stand of research describes how political skill can facilitate improved influence on formal policy and management processes. Whilst this study was concerned with studying the forms of political skill within the organisation of healthcare services, rather than more formal policy-making, the identified literature did suggest that political skill can extend beyond the organisational context and reach into more formal policy processes. Importantly, this highlights the important interface between healthcare policy making and healthcare organisations that is arguably less central to other business contexts and perhaps why it is little discussed in the wider literature. Of note here is the way that political skill, and its associated terms, have been used by the professions to advance their ideological agenda and professional power through influencing policy. This is exemplified by the seminal work of Clark [4] that set out an operational definition of nurses’ ‘political astuteness’ in terms of their participation in formal democratic voting processes, membership of political bodies, understanding of formal (US) political institutions and processes, and use of channels to engage in policy decision-making. This research seems concerned with advancing the professional status and position of nursing, vis a vis other professionals and groups in policy-making processes. Subsequent research has both developed and specified the work of Clark. For example, Byrd et al. [23] use the Political Astuteness Inventory to examine nursing students’ awareness of policy processes; whilst Benton et al. [30] describe nurses’ influence on policy-making as a form of ‘political competence’ including awareness of political processes and understanding of the procedures through which to influence legislative processes. Outside of nursing, Wilber and Coberly [59] examine gerontologists as professionals proficient in public policy, and advocate for more engagement in legislation and policy. Significantly, this literature talks of political skill as a form of ‘upward’ influence on formal policy processes, rather than influence in more local organisational processes, which is a marked difference to the studies discussed above.

Looking outside of professional domains, more recent research on major system change also describes the importance of system leaders having forms of political skill or intelligence through their awareness of and ability to navigate changing ‘top-down’ policy expectations that routinely influence efforts to reconfigure regional care services [58, 69, 71]. In particular, efforts to implement change within regional care systems can be facilitated through aligning with and using the expectations and financial inducements made available by national policy. Unlike the earlier literature, this research portrays political skill in terms of understanding of informal organisational or inter-organisational politics in the context of more formal national politics drivers.

## Discussion

Over the last four decades, concepts such as political astuteness, skill and savvy have been used to describe, or advocate for, the use of particular skills, behaviours and tactics when seeking to implement change in healthcare services. An initial point for discussion is that over this time, different professional and policy contexts have provided the impetus or backdrop for research. Defining political skill is therefore challenging. This review shows that the nursing field has provided the initial and main focus of health services research, including review articles summarising the influence of nurse leaders on policy-making and organisational change [5, 30]. By taking an inclusive approach that extended beyond a given clinical or professional domain, our review shows how political skill is also used by hospital managers and other health professionals when seeking to introduce organisational change, and, more recently, system leaders’ implementation of major system change. What seems to unite some of these studies (e.g. Montavlo [5, 44]; Montavlo and Byrne [25]; Berger [61]; Taylor [58]) is the idea that healthcare leaders need to develop and deploy political skill because of the perceived dominance of other healthcare professionals, who are often presented as resistant to change and having institutionalised power in the division of labour. However, more research is needed to understand the forms and use of political skill across the wider healthcare workforce and other stakeholders, including in different service settings and career stages.

The second point for discussion relates to the way political skill, or associated terms, have been conceptualised and deployed across the health services literature. To some extent, there is limited definitional or conceptual agreement. Much of the earlier research, especially in nursing, uses terms such as political astuteness and skill in largely descriptive and generalised ways drawn from observational studies and with limited theoretical underpinning. Subsequent use of these terms becomes infused with concepts and ideas drawn from management studies, but it is only later that the Ferris et al. [19] conceptualisation of political skill becomes the dominant framing for research [5]. We thus notice a shift from a relatively ‘loose’ descriptive concept to a ‘tighter’ analytical concept. As discussed below, whilst this shift results in enhanced clarity, it is also rigid and risks marginalising certain aspects in favour of others; notably the Ferris conceptualisation does not address leadership as an interpersonal process but rather focuses on individuals. The concept of political skill, as largely shaped by organisational psychology, speaks to the distinct capabilities and actions of engaging in organisational or micro-politics, in much the same way that broader concepts such as social skill or managerial skill, speak to the processes of engaging in field change or organisational change; where these are not regarded as mutually exclusive concepts but opportunities for varying levels and forms of analysis. The five aspects of political skill presented in this review (personal performance; contextual awareness; inter-personal influence; stakeholder engagement, networks and alliances; and policy influence) are directly comparable with the work of Ferris et al. [19], but they also highlight factors beyond individual skills in ways that places the individual within the wider organisational and system context of healthcare services, and public policy environment. Future research might therefore benefit from drawing upon alternate conceptualisations that are more relevant to issues of leadership and change in complex organisational contexts. For example, the conceptualisation of ‘political astuteness’ offered by Hartley [9] and Hartley and Bennington [10] not only deals directly with leadership, including healthcare leadership, but also describes skills, judgements and activities that are wider that interpersonal influence and which set these in the context of the demands of the job and the organisational context.

The third point for discussion addressed the variable levels of evidence underpinning these different dimensions and, more importantly, the relationships between these dimensions. Our review has focused on the organisational or micro-politics within healthcare services. As stated earlier, the micro-level analysis is concerned with the interactions between actors and groups, their differences and competing interests. Micro-politics is not limited to an organisational level, such as front line staff or executive board members, nor does it exclude macro-level institutional agendas or ideologies. This understanding of micro-politics stemming from macro-political interests is where our work arguably departs from the narrower psychological work on organisational politics and re-engages with the wider political theory. Where one foregrounds agency and behaviours the latter emphasises structures and ideologies. Based on our findings, we argue for attending to both, and the interplay between the two, in a non-reductionist sense.

The review finds there is more descriptive evidence on the importance of leaders’ ‘contextual awareness’ of prevailing lines of power, and in more general ways, the importance of ‘stakeholder engagement’ and effective ‘communication’. In other areas, however, the evidence-base appears relatively thin and under-developed. Although ‘inter-personal influence’ features across much of the literature, there is surprisingly little analytical detail how what form or impact this takes beyond relatively general accounts of negotiation, persuasion and coercion. For example, how is persuasion realised through the use of evidence or the use of incentives and sanctions. Moreover, there is little detail of how such inter-personal influence varies between occupational groups and organisational contexts. As discussed below, much of the review literature describes political skill in terms of individual or personal qualities, rather than necessarily professional or organisational in character.

The existing literature rarely explores the inter-connections between the different aspects of political skill. For instance, individuals’ use of ‘stakeholder engagement’ appears to be collapsed alongside building ‘networks and alliances’; however these might better be thought of as two related, but distinct, processes that require different skills. Even those studies drawing explicitly on the framework of Ferris et al. [19] tend to treat the different dimensions of political skill as relatively discrete variables with only a small number of papers elaborating the inter-connections between the constituent parts of political skill. One example being Montalvo’s [5] integrative review that shows political skill as operating first through ‘the self’, then through influence on ‘others’ and then on the performance of the ‘organisation’.

Thus, we recognise that organisational politics can encompass relatively narrow or self-serving interests as often depicted in the organisational psychology literature, but also deeper or broader interests associated for example with professional power in the division of labour or broader political governing rationalities manifest in social discourse. As such, the interests driving organisational politics can be seen as operating on multiple dimensions simultaneously. This can include highly personal interests for career advanced or organisational priorities for improved efficiencies or quality, and may also reflect deeper professional agenda or structural interests around challenging dominant groups or maintaining the status quo; further still these can reflect deeper ideological imperatives around the social value of care. When individuals and groups interact around a change initiative it is important to see the politics and political skill as motivated by these multiple intertwined interests, some of which will be espoused, some hidden and other subconscious, so that the implementation of change may be presented as advancing some social good, but at the same time enhancing the reputation of the change leader or advancing the shared m agenda of a profession and also reflecting deeper ideological assumptions about contemporary public service.

**Fig. 2:**
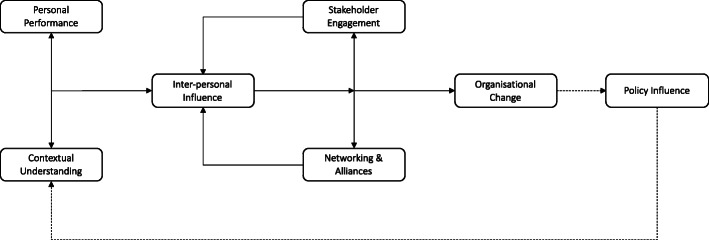
Conceptual heuristic of political skill ‘in action’

Through our review of the health service research literature, we sketched out the foundations for a more integrative conceptual heuristic (Fig. 2).

Specifically, extant literature suggests a close connection or interplay between ‘personal performance’ and ‘contextual awareness’, in that leaders need to effectively understand their own skills and capabilities relative to the political landscape, lines of power and systems of influence. This provides the orientation and basis for ‘inter-personal influence’, in terms of understanding who to influence and how to influence them, given the leader’s given position and skills. In turn, such influence extends from the individual to the group level, to include ‘stakeholder engagement’ and in parallel ‘network building’ which provides the basis for coordinated and collective ‘organisational change’, often in the context of resistance or opposition. Moreover, stakeholder engagement and networking and alliances can provide positive feedback loops for enhancing inter-personal influence with other stakeholders. Finally, and somewhat tangentially, the literature also suggests leaders can also seek to use political skill to engage in more formal ‘policy influence’; perhaps to reinforce the more informal forms of political skill. We also argue that in light of the findings that do discuss policy, it is important for leaders to be aware of and consider any policy influences within their wider context.

As indicated above, the shift towards a tighter and more specific conceptualisation of political skill affords more comparative and analytical sophisticated research, but also emphasises certain dimensions of organisational politics and political skill to the exclusion of others. The influence of Ferris and colleagues might offer a more precise conceptualisation, but by drawing primarily on social psychology, this presents a highly individualised and behavioural understanding of political skill. Such a conceptualisation is associated with a person’s psychological qualities to read people and situations, their behavioural skills and competencies to influence others, and their self-reflective skills to assess their own behaviours in relation to others. While this may include consideration of an actor’s inter-personal relations, the underlying assumption is that individuals are able to navigate conflict or politics in the workplace in pursuit of relatively narrow personal motives and organisational agendas. This approach underpins a major strand in contemporary leadership development that encourages leaders and aspirant leaders to reflect upon their psychological qualities and fine-tune their inter-personal skills to influence others. In contrast, there remains a substantial body of health services research that does not use the term or concept of political skill (or associated terms), and hence was not included in this review; but importantly this alternate literature analyses the micro-politics of health service organisation in ways that goes beyond individual skills and behaviours. This includes, for example, a long tradition of research on the negotiated order of healthcare organisation [83, 84]. In addition, more critical and interpretative perspectives on micro-politics bring to the fore the influence of prevailing institutional and ideological forces on the local manifestations of change, such as the divergent agendas around professionalism and managerialism [2]. Importantly, this literature demonstrates the importance of looking beyond individual skills and capabilities to understand the social and cultural context of ‘political action’ together with more explicit recognition of the structural inequalities that frame organisational politics related, for instance, to issues such as profession, gender, ethnicity or class. Bridging the conceptual divide between the mainstream health services research literature deploying the concept of political skill and this more critical sociological literature offers extended insights for examining political skills and practices in the context of wider social and political forces. The heuristic we derive from the literature points to the complex interplay between actors, context and processes of change, but so far there has been little explicit research which tries to examine these factors within specifically designed studies.

There are lessons arising from this research for implementing healthcare change. As we have argued above, conceptualisations of political skill should include individual qualities, wider structural, cultural and ideological issues, and processes of change, along with the interplay between them. This is especially important, as rather than a framework of what works well in what context, it is clear from the literature that effectively implementing change requires leaders to move rapidly and simultaneously from the level of individuals, groups, structures and policies, whilst also remaining sensitive to their context and power dynamics. Treating each of these as separate entities or having a tight framework of success criteria significantly weakens a leader’s ability to implement change. It is the fluidity between these, and the ability to reflect, process changes and adapt strategies that is a central component to implementing healthcare change. Such attention enables leaders to effectively exercise their influence on the individual and group level, knowing who to influence, how and in what contexts.

### Limitations

There are inevitable limitations to this review. Because of the wide variety of terms used for political skill, and how it is often incorporated within wider leadership literature, it is possible that some papers may have been missed that use un-specified terms. Specifically, some healthcare studies and literature may describe organisational phenomena that is similar to political skill, but it is not labelled as ‘political’ and therefore not captured in our search strategy. A parallel limitation relates to the boundaries between health services research and related disciplinary fields. For example, research within the management studies fields might use healthcare organisations as one of multiple case study sites to compare leadership differences, but with limited detailed account of the health service context. In this review, the researchers discussed the inclusion of such papers based upon the detail and novelty of conceptualization and application, but again there is scope that some studies could have been excluded. Further, the review is limited by the quality and quality assessment of empirical papers, especially as many of the included studies do not define the concept of political skill and were vague with their methodological approaches. A more significant limitation of the review process is the exclusion of many seminal and influential texts in the field of healthcare policy and politics, such as Alford’s [85] *Healthcare Politics* or Klein’s [82] *The New Politics of the National Health Service*. Although service change does involve policy, these works informed the framing and initial specification of this research they were excluded on the grounds that they rarely, if ever, explicitly talk of political skill or related terms, even though they focus on the manifestation of broader political interests and process on the everyday organisation of care. Further research could include an analysis of the use of political skill in relation to policy. A number of other prominent commentaries and essays were identified that talked at length about the concept of political skill and astuteness [86, 87] but were excluded because they were primarily review essays and offered limited detail or empirical substance. Lastly, there were some papers that discussed system change (e.g. Maun et al. [88]), but did not discuss political skill or its related terms. As we discuss above, future research building upon these concepts could determine the extent to how they influence organisational change.

## Conclusion

This review identifies that health service leaders’ use of political skill in the implementation of change arises and operates within five thematic dimensions, namely: personal performance; contextual awareness; inter-personal influence; stakeholder engagement, networks and alliances; influence on policy processes. Going forwards, engaging stakeholders and building networks and alliances could be more usefully conceptualised as distinct, but related, processes that influence organisational change. More significantly, the review shows how these dimensions can be inter-connected to accumulate influence in the context of prevailing lines of power in the organisation of care. These dimensions can be instructive to the development of current and future healthcare leadership programmes that seek to support healthcare leaders better understand and navigate the organisational politics of healthcare services. The review also highlights that the evolution of the empirical and conceptual research over the last four decades has led to a view of political skill as highly individualised and based on psychological attributes to the neglect of more social skills or collective actions undertaken in the context of wider social, cultural or political factors. As such, future research could better understand and evidence these additional factors to offer a more developed and social understanding of how political skill contributes to the implementation of health services change.

## Supplementary Information

**Additional file 1.** Search Strategy.

## Data Availability

The datasets used and/or analysed during the current study are available from the corresponding author on reasonable request.

## Abbreviations

IT: Information technology
UK: United Kingdom
