# Supplementary material for: The contribution of political skill to the implementation of health services change: a systematic review and narrative synthesis
Source: BMC Health Serv Res. 2021 Mar 20;21:260. doi: 10.1186/s12913-021-06272-z (PMC7981881; doi:10.1186/s12913-021-06272-z)
Supplement: Supplementary file 1 — Additional file 1. Search Strategy. [file 12913_2021_6272_MOESM1_ESM.docx]

**Appendix 1 Search Strategy**

*PubMed (https://pubmed.ncbi.nlm.nih.gov/)*

Title and abstract search

1. Politic* astute*
2. Politic* Savv*
3. Politic* Acume*
4. Politic* Nous*
5. Politic* Antennae*
6. Politic* Skill*
7. Socio-politic* Intelligen*
8. Politic* leadership*
9. 1 or 2 or 3 or 4 or 5 or 6 or 7 or 8
10. health*
11. health* service*
12. health* system*
13. health* policy
14. health* policies
15. healthcare*
16. 10 or 11 or 12 or 13 or 14 or 15
17. Large scale*
18. Major system*
19. Whole system*
20. Organisation* change*
21. Inter-organisation* change*
22. 17 or 18 or 19 or 20 or 21
23. 9 and 16 and 22

Results = 74 (limited to full text, English)
